# Supplementary material for: The T Cell Receptor (TRB) Locus in Tursiops truncatus: From Sequence to Structure of the Alpha/Beta Heterodimer in the Human/Dolphin Comparison
Source: Genes (Basel). 2021 Apr 14;12(4):571. doi: 10.3390/genes12040571 (PMC8070946; doi:10.3390/genes12040571)

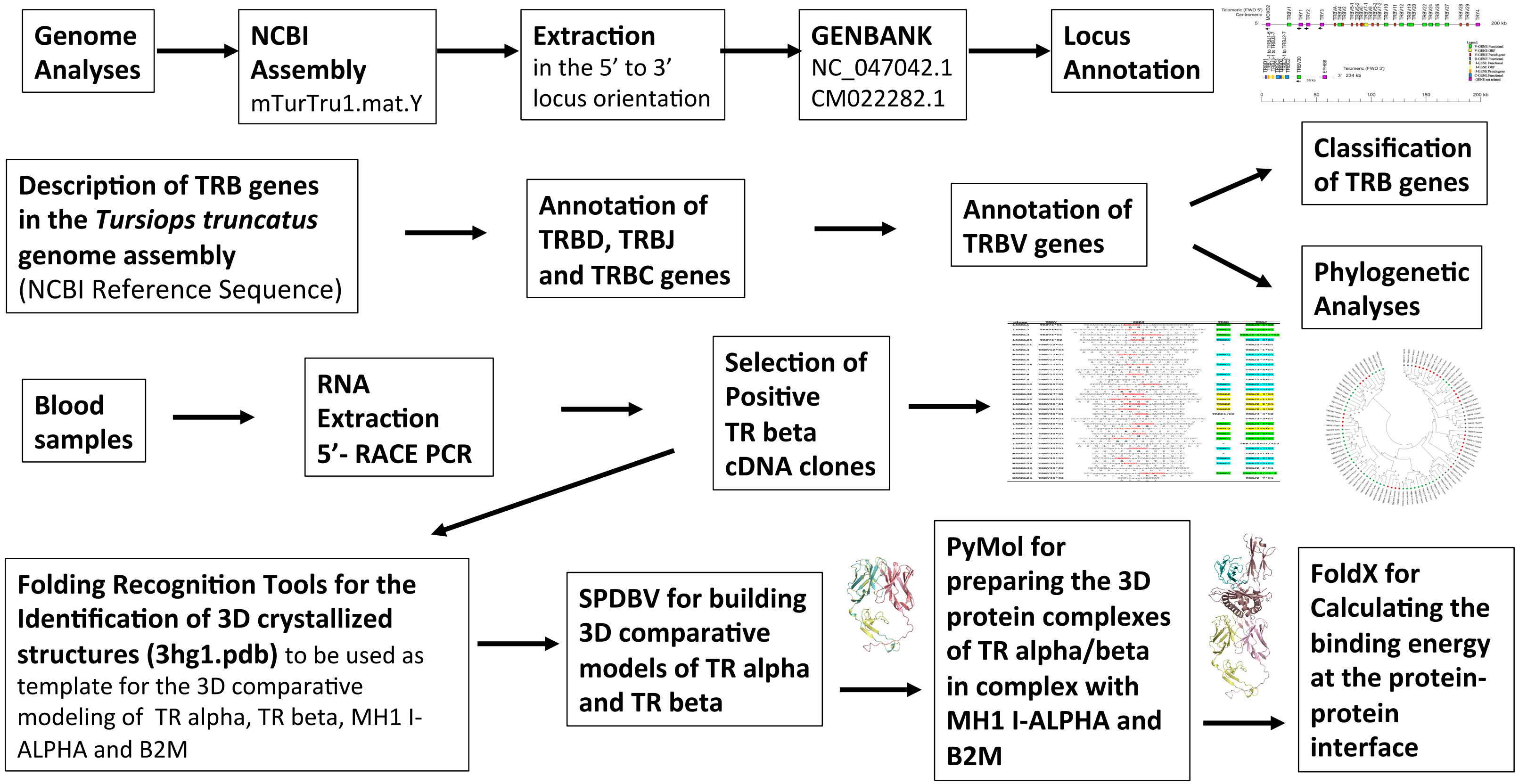

Figure S1. Flowchart that depicts the overall experimental setting and analysis.

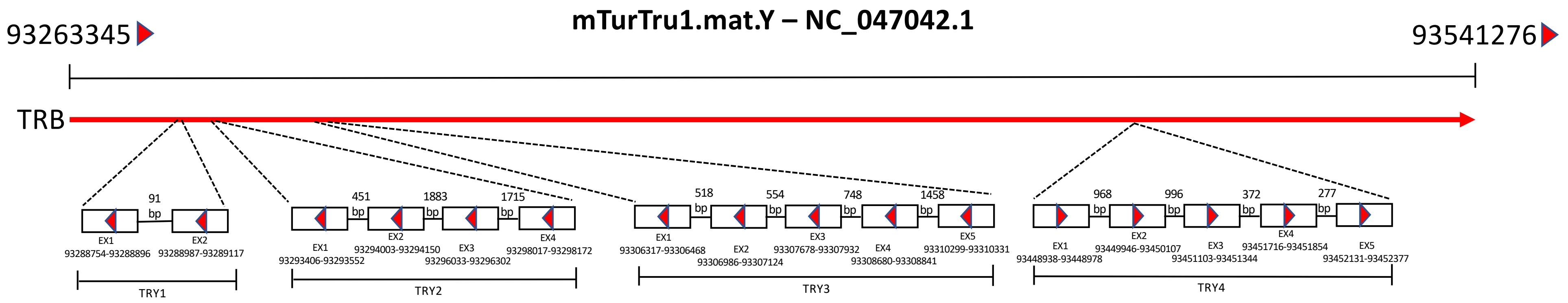

Figure S2. Structure of the dolphin trypsin-like serine protease (TRY) genes within TRB locus (NC_047042.1). TRY1, TRY2 and TRY4 are partially annotated. The exons are represented with boxes and their positions within the NC_047042.1 sequence are reported. The red arrowheads indicate the transcriptional orientation of the genes.

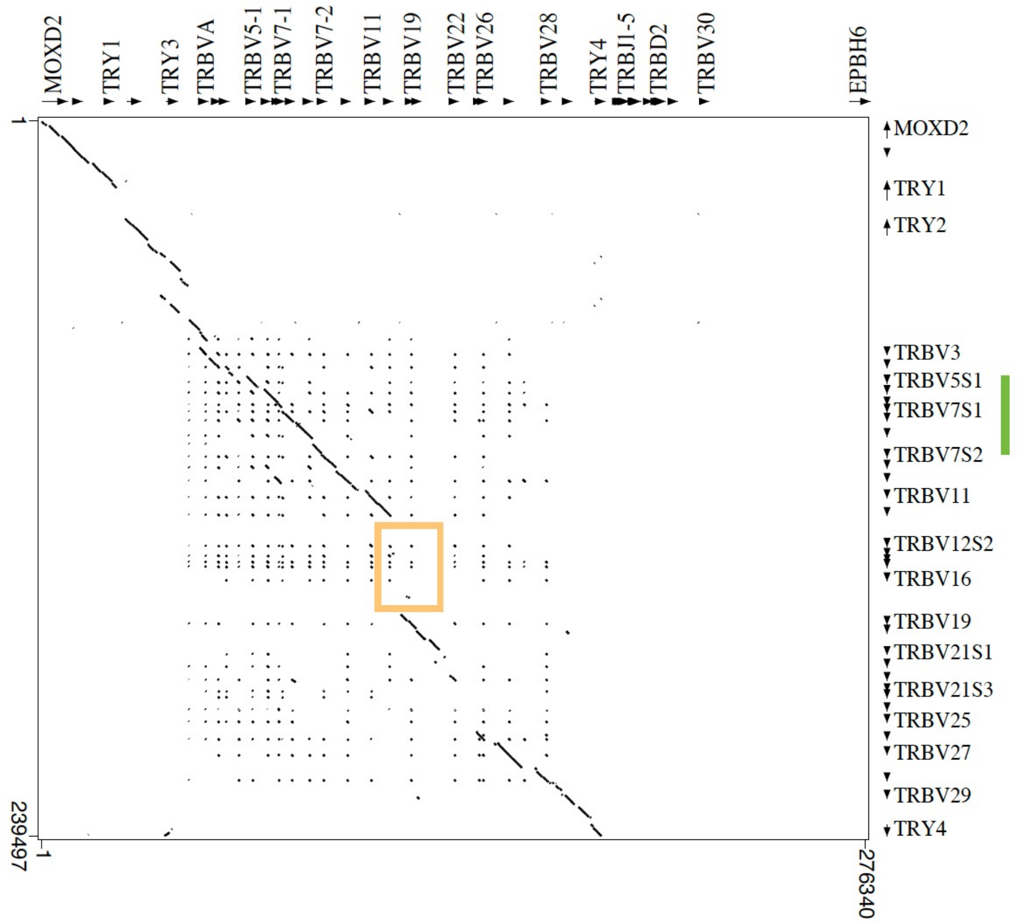

Figure S3. Dot-plot matrix of dolphin/dromedarius TRB sequence genomic comparison. The transcriptional orientation of each gene is indicated by arrow heads. Colored rectangle (orange) encloses TRBV deleted regions in dolphin as referred in the text. The green line indicates the only region where gene duplication took place in *T. truncatus* TRB locus.

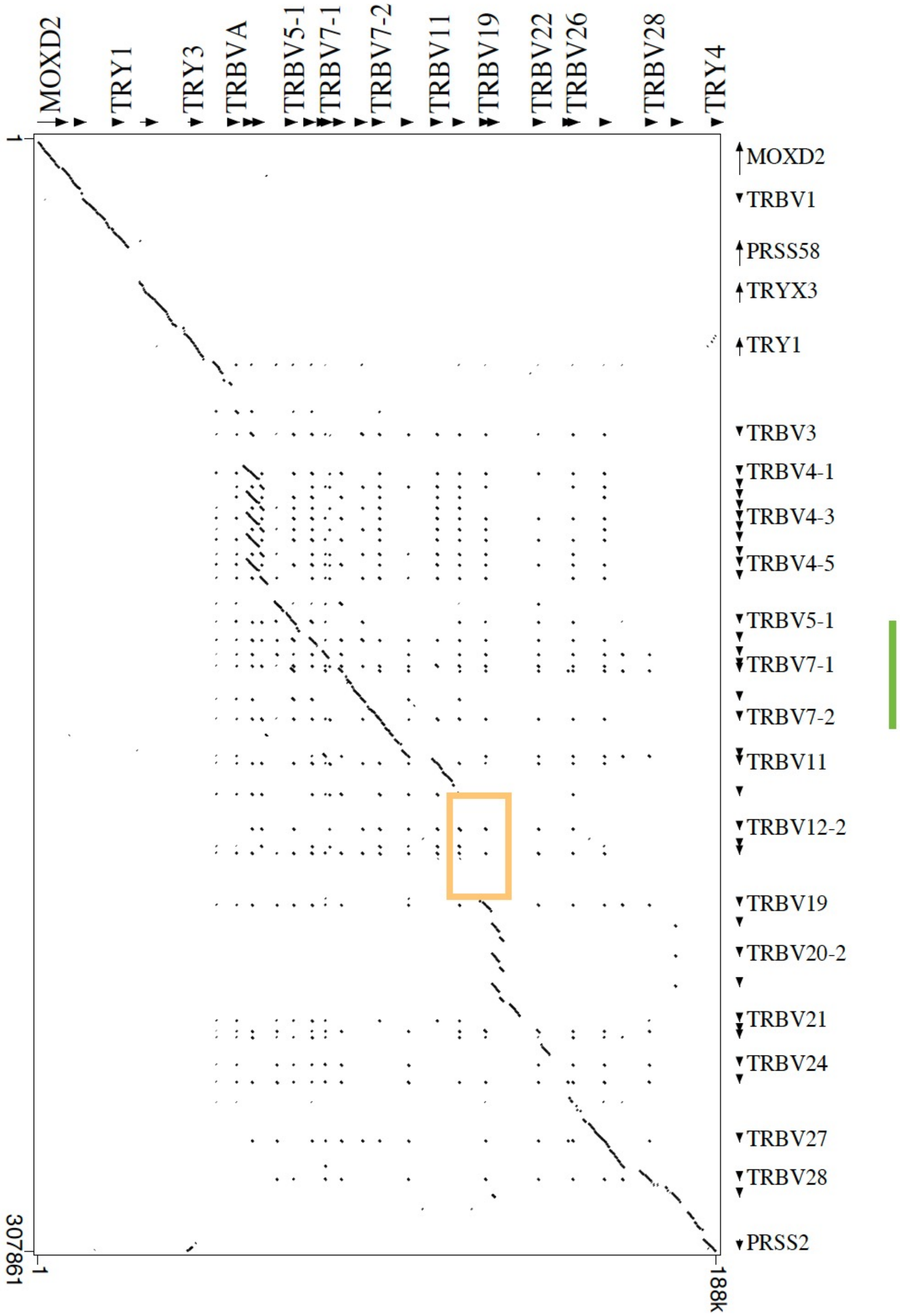

Figure S4. Dot-plot matrix of dolphin/pig TRB sequence genomic comparison. The transcriptional orientation of each gene is indicated by arrow heads. Colored rectangle (orange) encloses TRBV deleted regions in dolphin as referred in the text. The green line indicates the only region where gene duplication took place in *T. truncatus* TRB locus.

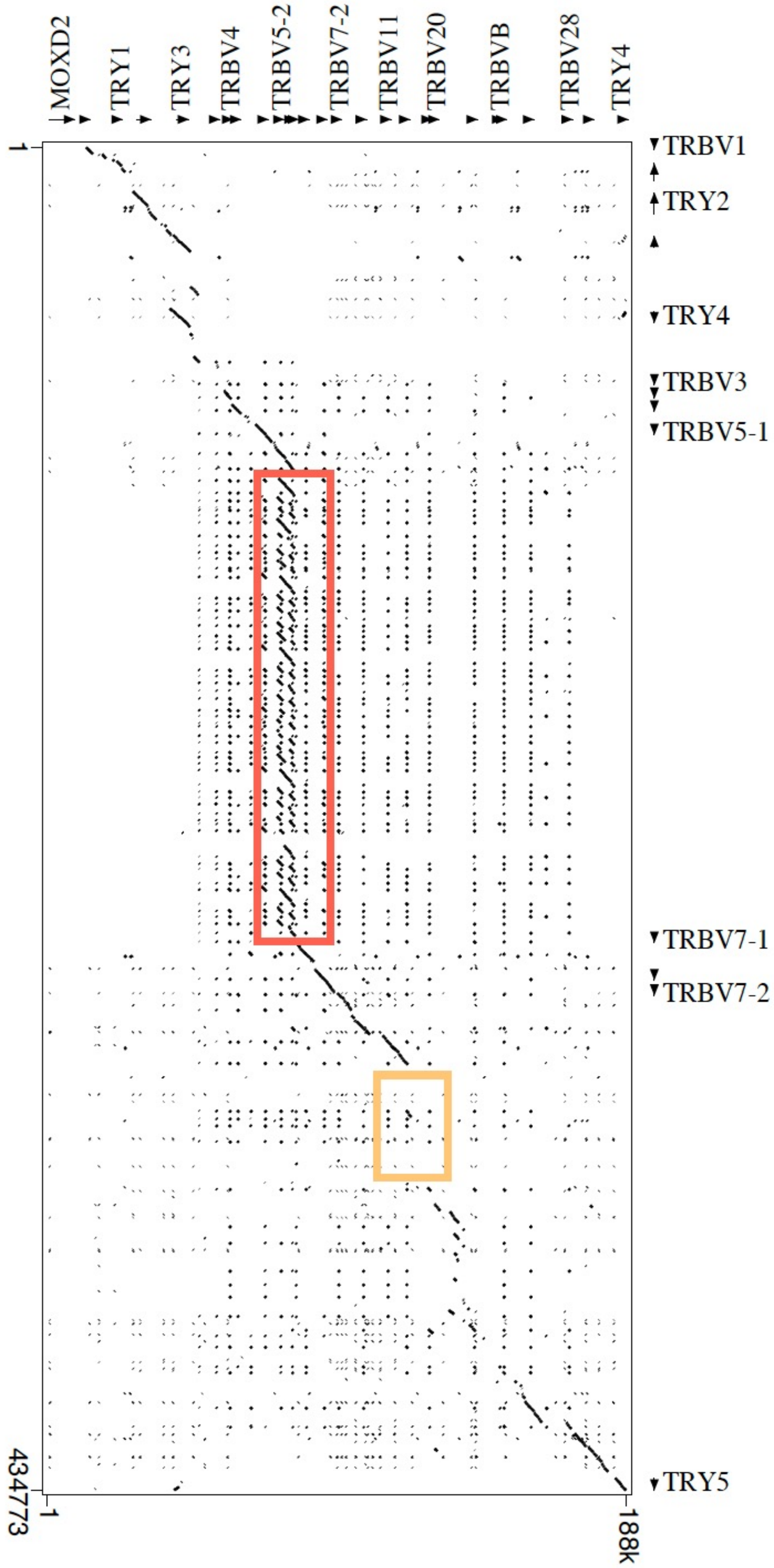

Figure S5. Dot-plot matrix of dolphin/goat TRB sequence genomic comparison. The transcriptional orientation of each gene is indicated by arrowheads. Colored rectangles (red) enclose TRBV duplicated regions in goat and (orange) TRBV deleted regions in dolphin as referred in the text.

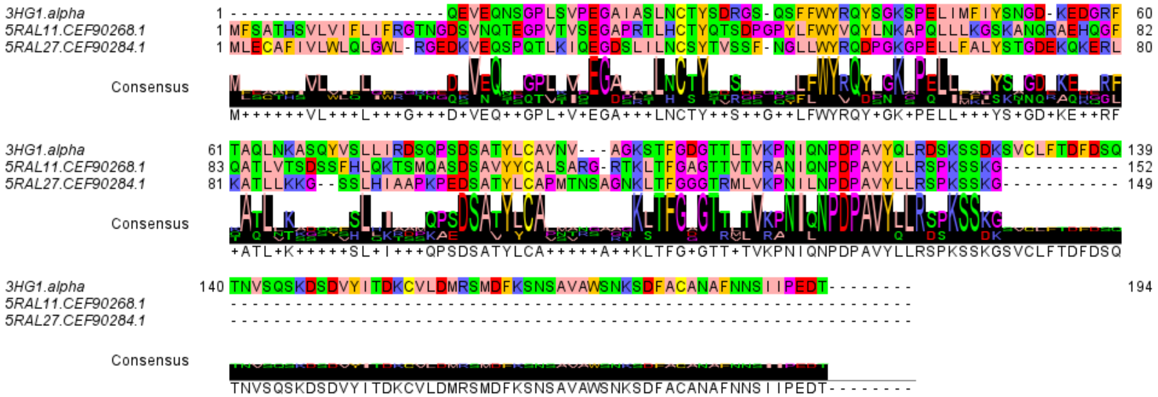

Supplementary Fig. S6 A. Sequence-structure alignment of the *T.truncatus* TR alpha chains with human TR alpha chains from the crystallized structures 3hg1.pdb and 5d2l.pdb

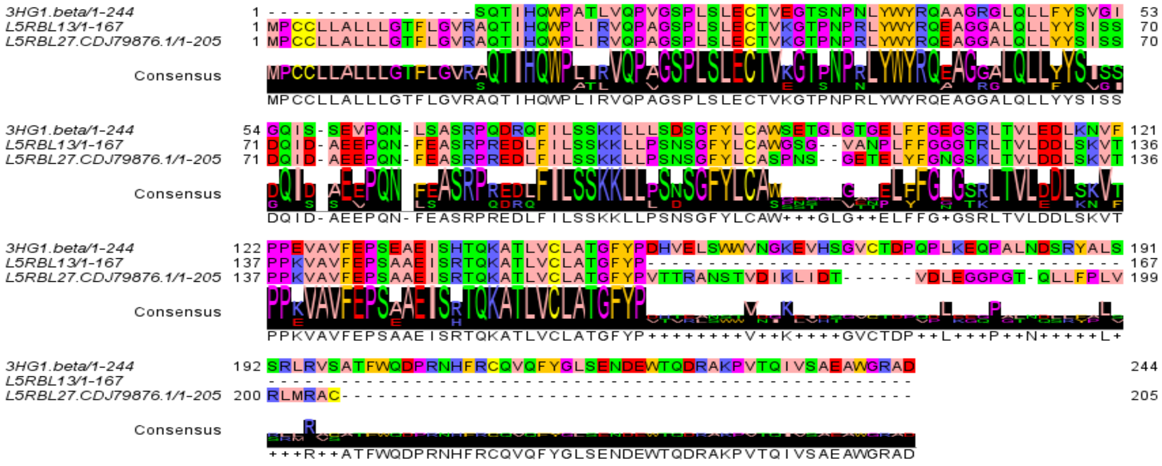

Supplementary Fig. S6 B. Sequence-structure alignment of the *T.truncatus* TR beta chains with the human TR beta chains from the crystallized structures 3hg1.pdb and 5d2l.pdb

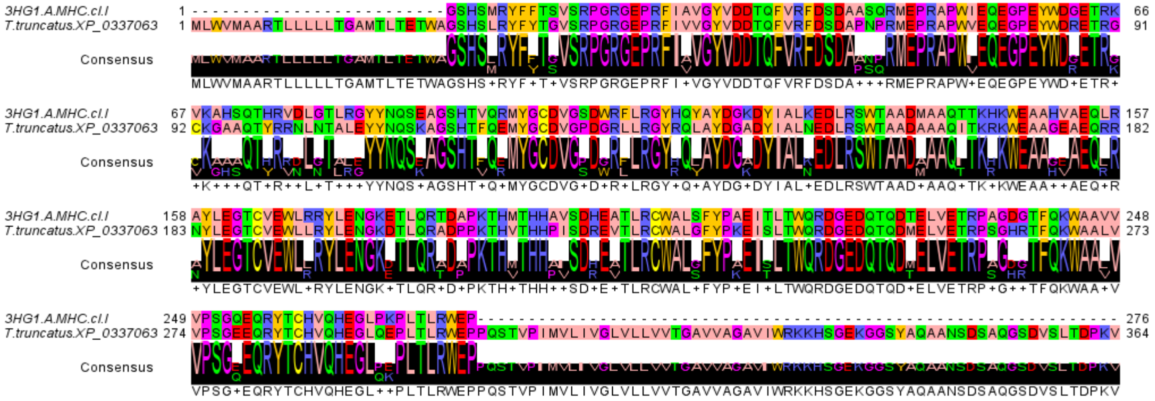

Supplementary Fig. S6 C. Sequence-structure pairwise alignment of the human MH1 I-ALPHA (from 3hg1.pdb) with its closest counterpart in *T.truncatus*

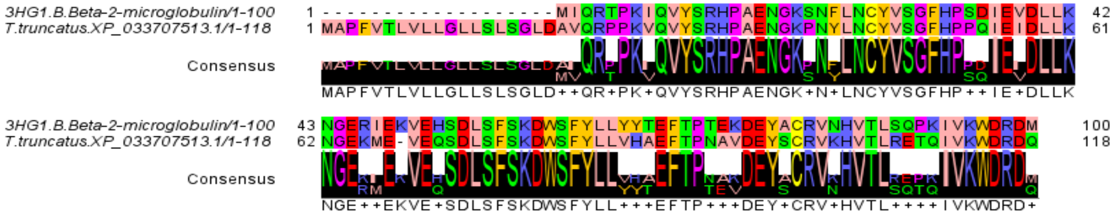

Supplementary Fig. S6 D. Sequence-structure pairwise alignment of the human B2M (from 3hg1.pdb) with its closest counterpart in *T.truncatus*

Supplementary Table S1. Description of the TRB genes in the *Tursiops truncatus* genome assembly NC_047042.1 (NCBI Reference Sequence). The exon-intron positions of all genes, their classification and functionality are reported.

TRBV subgroup	TRBV gene name	Functionality	L-Part1 (Positions in NC_047042.1)	Intron (Positions in NC_047042.1)	V-exon (Positions in NC_047042.1)
TRBVA	TRBVA	P	93320055-93320103	93320104-93320206	93320207-93320358
TRBV1	TRBV1	F	93278021-93278075	93278076-93278358	93278359-93278653
TRBV2	TRBV2	P	93326918-93326963	93326964-93327049	93327050-93327347
TRBV4	TRBV4	F	93324321-93324369	93324370-93324481	93324482-93324776
TRBV5	TRBV5-1	P	93335335-93335383	93335384-93335502	93335503-93335774
	TRBV5-2	P	93340222-93340270	93340271-93340398	93340399-93340693
	TRBV5-3	P	93354230-93354278	93354279-93354429	93354430-93354715
TRBV6	TRBV6	P	93343983-93344031	93344032-93344076	93344077-93344322
TRBV7	TRBV7-1	P	-	-	93345165-93345463
	TRBV7-2	P	93358991-93359038	93359039-93359162	93359163-93359460
TRBV8	TRBV8	ORF	93348343-93348388	93348389-93348519	93348520-93348814
TRBV10	TRBV10	F	93366739-93366787	93366788-93366879	93366880-93367174
TRBV11	TRBV11	P	93374729-93374779	93374780-93374901	93374902-93375179
TRBV12	TRBV12	F[b]	93380788-93380830	93380831-93380921	93380922-93381219
TRBV19	TRBV19	F	93387915-93387963	93387964-93388095	93388096-93388390
TRBV20	TRBV20	F	93389954-93389984	93389985-93390300	93390301-93390604
TRBV22	TRBV22	F	93401774-93401822	93401823-93401940	93401941-93402229
TRBV24	TRBV24	F	93407335-93407383	93407384-93407511	93407512-93407806
TRBV26	TRBV26	F	93413510-93413558	93413559-93413663	93413664-93413958
TRBV27	TRBV27	F	93422186-93422243	93422244-93422369	93422370-93422664
TRBV28	TRBV28	P	93434684-93434726	93434727-93434562	93434563-93434857
TRBV29	TRBV29	P	93440755-93440788	93440789-93441056	93441057-93441360
TRBV30	TRBV30	F	93487190-93487492	93487493-93487826	93487827-93487870

[b] STOP-CODON at position 108 (last 3' codon of germline CDR3-IMGT) may disappear during rearrangements

TRBD gene name	Functionality	Positions in NC_047042.1
TRBD1	F	93457912-93457924
TRBD2	F	93471630-93471644

**TRBJ gene name**	**Functionality**	**Positions in NC_047042.1**
TRBJ1-1	F	93458569-93458616
TRBJ1-2	ORF	93458704-93458747
TRBJ1-3	F	93458981-93459030
TRBJ1-4	ORF	93459601-93459649
TRBJ1-5	P	93459873-93459894
TRBJ1-6	F	93460364-93460416
TRBJ3-1	F	93463121-93463170
TRBJ3-2	ORF	93463316-93463366
TRBJ3-3	F	93463536-93463584
TRBJ3-4	F	93463682-93463730
TRBJ3-5	F	93463804-93463849
TRBJ3-6	P	93463900-93463950
TRBJ3-7	F	93464105-93464151
TRBJ2-1	F	93472292-93472342
TRBJ2-2	F	93472489-93472536
TRBJ2-3	F	93472706-93472754
TRBJ2-4	F	93472849-93472897
TRBJ2-5	F	93472971-93473015
TRBJ2-6	F	93473066-93473118
TRBJ2-7	F	93473285-93473331

**TRBC gene name**	**Functionality**	**EX1 (Positions in NC_047042.1)**	**INT1 (Positions in NC_047042.1)**	**EX2 (Positions in NC_047042.1)**	**INT2 (Positions in NC_047042.1)**	**EX3 (Positions in NC_047042.1)**	**INT3 (Positions in NC_047042.1)**	**EX4 (CDS) (Positions in NC_047042.1)**	**EX4 (3'UTR) (Positions in NC_047042.1)**
TRBC2	F	93475738-93476124	93476125-93476824	93476825-93476842	93476843-93477013	93477014-93477120	93477121-93477399	93477400-93477417	93477418-93479542
TRBC3	F	93467445-93467831	9346446-93468532	93468533-93468550	93468551-93468721	93468722-93468828	93468829-93469147	93469148-93469165	93469166-93469864

Supplementary Table S2. Description of the unrelated TRB genes in the *Tursiops truncatus* genome assembly NC_047042.1 (NCBI Reference Sequence). The position of all genes and their classification and functionality are reported.

Gene classification	Functionality	Position
MOXD2	F	93263345-93275829
TRY1	n.d.	93288754-93289117
TRY2	n.d.	93293406-93298172
TRY3	F	93306317-93310331
TRY4	n.d.	93448938 93452377
EPHB6	F	93534281-93541276

Supplementary Table S3 . GEDI accession numbers for TRBV genes of *Camelus dromedarius, Sus scrofa, Capra hircus and Tursiops truncatus.* The position of all genes and their functionality are reported.

Species	Genes	Functionality	GEDI* databases accession number	Position	References
Camelus dromedarius	TRBV1	F	NW_011591622	861263-861886	[26] doi: 10.1016/j.dib.2017.08.002<br><br>[27] doi: 10.1016/j.molimm.2007.05.023
	TRBV2	F		932263-932714	
	TRBV3	P		927952-928412	
	TRBV5S1	F		937384-937843	
	TRBV6	F		944809-945237	
	TRBV7S1	F		947134-947581	
	TRBV8	F		950124-950593	
	TRBV9	P		965923-966346	
	TRBV10	F		970368-970809	
	TRBV11	F		975860-976308	
	TRBV12S1	P		981727-982197	
	TRBV14	P		995472-995906	
	TRBV15S1	F		997569-998023	
	TRBV16	F		1003645-1004098	
	TRBV19	F		1018094-1018641	
	TRBV20	F		1020910-1021565	
	TRBV21S1	F		1028337-1028797	
	TRBV22	F	NW_011591151	46518-46381	
	TRBV23	P		60590-60480	
	TRBV24	P		56428-56106	
	TRBV25	F		52347-52219	
	TRBV26	F		66428-66297	
	TRBV27	F		41158-41032	
	TRBV28	F		32762-32640	
	TRBV29	F		27109-26837	
	TRBV30	F	NW_011593440	14509-14160	
Sus scrofa	TRBV1	F	NC_010460	7734192-7733565	[24] doi: 10.3389/fimmu.2018.02526
	TRBV2-1	P		7655012-7642779	
	TRBV3	F		7669490-7669041	
	TRBV4-1	F		7658715-7658261	
	TRBV5-1	F		7617595-7617140	
	TRBV6	P		7608622-7608194	
	TRBV7-1	F		7605421-7604971	
	TRBV8	P		7604130-7603671	
	TRBV10	F		7580532-7580066	
	TRBV11	F		7578532-7578085	
	TRBV12	F		7569942-7569498	
	TRBV14	F		7555547-7555118	
	TRBV15	F		7553638-7553174	
	TRBV19	F		7539369-7538893	
	TRBV20-1	F		7533974-7533275	
	TRBV21	F		7507323-7506866	
	TRBV22	P		7504424-7503959	
	TRBV23	P		7502722-7502260	
	TRBV24	F		7495123-7494651	
	TRBV25	F		7490331-7489856	
	TRBV27	F		7474142-7473643	
	TRBV28	P		7463497-7462998	
	TRBV29	F		7459048-7458439	
	TRBV30	F		7397804-7398474	
Capra hircus	TRBV1	F	NC_030811.1	14984015-14984322	[32] doi: 10.1186/s12864-020-07022-x
	TRBV2	F		14898809-14899260	
	TRBV3	F		14907378-14907838	
	TRBV4	F		14903033-14903488	
	TRBV5-2	F		14884857-14885334	

	TRBV6-1	F		14880001-14880431	
	TRBV7-1	F		14726886-14727332	
	TRBV12-1	F		14687500-14687943	
	TRBV14-1	P		14669550-14669978	
	TRBV15	F		14667225-14667689	
	TRBV16	F		14662154-14662607	
	TRBV19	F		14646735-14647211	
	TRBV20	F		14641339-14642152	
	TRBV21-1	F		14634677-14635133	
	TRBV22	F		14601178-14601645	
	TRBV24	F		14595644-14596117	
	TRBV25	F		14589280-14589750	
	TRBV26	F		14587349-14587828	
	TRBV27	P		14575967-14576435	
	TRBV28	F		14569871-14570543	
	TRBV29	F		14562392-14562986	
	TRBV30	F		14503203-14503862	
Tursiops truncatus	TRBVA	P	NC_047042.1	93320055-93320358	This work, (Table Supplementary S1)
	TRBV1	F		93278021-93278653	
	TRBV2	P		93324321-93324776	
	TRBV4	F		93326918-93327347	
	TRBV5-1	P		93335335-93335774	
	TRBV5-2	P		93340222-93340693	
	TRBV5-3	P		93343983-93344322	
	TRBV6	P		93345165-93345463	
	TRBV7-1	P		93348343-93348814	
	TRBV7-2	P		93354230-93354715	
	TRBV8	ORF		93358991-93359460	
	TRBV10	F		93366739-93367174	
	TRBV11	P		93374729-93375179	
	TRBV12	F		93380788-93381219	
	TRBV19	F		93387915-93388390	
	TRBV20	F		93389954-93390604	
	TRBV22	F		93401774-93402229	
	TRBV24	F		93407335-93407806	
	TRBV26	F		93413510-93413958	
	TRBV27	F		93422186-93422664	
	TRBV28	P		93434684-93434857	
	TRBV29	P		93440755-93441360	
	TRBV30	F		93487190-93487870	

* GEDI (for GenBank/ENA/DDBJ/IMGT/LIGM-DB)

Supplementary Table S4. Description of the *Turtru* TRBV pseudogenes

TRBV genes	No INIT-CODON	Defective Leader	Frameshift	Stop codon
TRBVA	●	●	●	
TRBV2	●	●	●	
TRBV5-1			●	●
TRBV5-2		●	●	
TRBV5-3				●
TRBV6			●	
TRBV7-1		●	●	
TRBV7-2			●	●
TRBV11			●	●
TRBV28				●
TRBV29	●	●	●	

Supplementary Table S5. Contact analysis 3Hg1.

Homo sapiens V-ALPHA [6.6.9] TRAV12-2*01 (98.9%) -TRAJ27*01 (100%),
Homo sapiens V-BETA [6.5.13] TRBV30*01 (100%)-(TRBD) - TRBJ2-2*01 (100%)
HLA-A*0201 (100%) G-ALPHA1 (1-90) – G-ALPHA2 (91-182)- C-LIKE (183-274)
Peptide MART-1 10-mer ELAGIGILTV

V-ALPHA [6.6.9]		G-ALPHA1	Atom pairs contact types Total : 81											
		58E (1)	61D (1)	62G (17)	65R (48)**	66K (14)**								
CDR1-IMGT	37 Q					66K (3)								
CDR3-IMGT	109 A	58E (1)	61D (1)	**62G** (15)	**65R** (17)	66K (4)								
	114 G			62G (2)	**65R** (12)	66K (7)								
	115 K				**65R** (19)									
V-BETA [6.5.13]		G-ALPHA1	Atom pairs contact types Total : 100											
					65R (18)**	66K (6)**	68K (9)	69A (13)	70H (2)	72Q (30)	73T (10)	75R (4)	76V (8)	
CDR1-IMGT	36 N												76V (5)	
FR2-IMGT	55 Y				65R (7)			69A (1)						
CDR2-IMGT	57 V						68K (9)	69 A (3)		**72Q** (18)				
	58 G									72Q (6)				
FR3-IMGT	66 Q									72Q (5),		75R (4)		
	70 E				**65R** (11)									
CDR3-IMGT	109 T									72Q (1),	73T (4)		76V (3)	
	110 G							69A (4)			73T (5)			
	111 L					66K (6)		69A (5)	70H (2)		73T (1)			
V-ALPHA [6.6.9]		G-ALPHA2	Atom pairs contact types Total : 109											
				62H (4)	65E (13)	66Q** (29)		69A (8)	70Y (10)		73T (17)		76E (11)	77W (17)
CDR1-IMGT	27 D													77W (2)
	28 R										73T (1)		**76E** (11)	77W (7)
	29 G										73T (1)			77W (8)
	36 S										73T (1)			
	37 Q								**70Y** (10)		**73T** (13)			
CDR2-IMGT	57 Y			62H (4)	**65E** (13)	**66Q** (29)		69A (7)						
	58 S							69A (1)						
FR3-IMGT	82 K										73T (1)			
V-BETA [6.5.13]		G-ALPHA2	Atom pairs contact types Total : 24											
						66Q**								
CDR3-IMGT	112 G					66Q (9)								
	113 T					**66Q** (15)								

G-ALPHA1 and G-ALPHA2 amino acids which have atom pair contacts with both V-ALPHA and V-BETA are underlined in the alignments.
They include, in the G-ALPHA1:
65R (contacts with V-ALPHA 109A, 114G and 115K* and V-BETA 55Y and 70E*)
66K (contacts with V-ALPHA 37Q, 109A and 114G* and V-BETA 111L*)
and in the G-ALPHA2:
66Q (contacts with V-ALPHA 57Y*) and V-BETA 112G and 113T*).

Dataset S1. Schematic representation (20X zoom) of the *Tursiops truncatus* TRB locus D-J-C region deduced from the genome assembly mTurTru1.mat.Y (CM022282.1). The boxes representing the genes are not to scale. The exons are not shown.

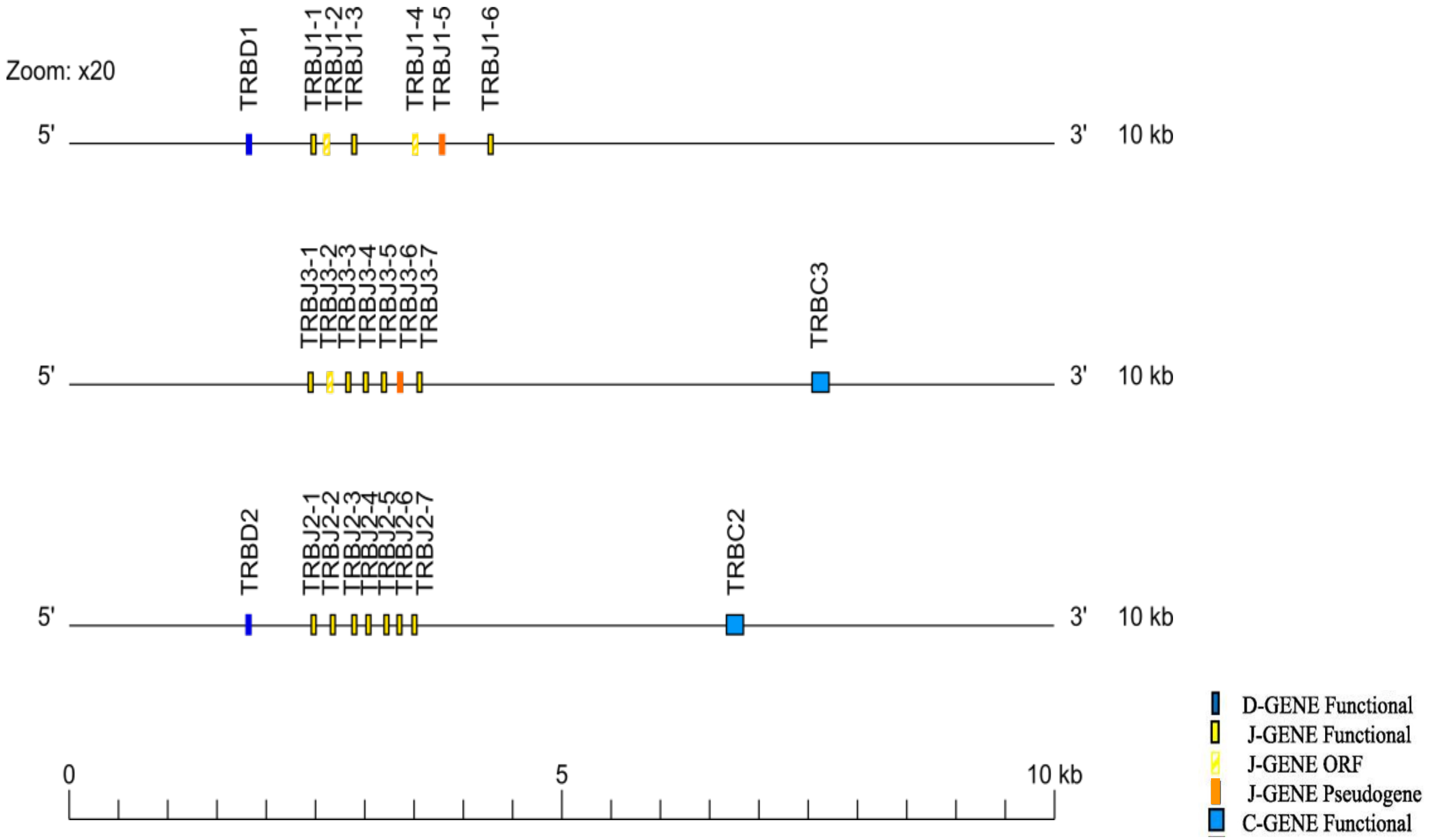

Supplement: Supplementary file 1 [file genes-12-00571-s001.pdf]
